# Supplementary material for: Overcoming structural violence through community-based safe-spaces: Qualitative insights from young women on oral HIV pre-exposure prophylaxis (PrEP) in Kisumu, Kenya
Source: PLOS Glob Public Health. 2025 Feb 24;5(2):e0004220. doi: 10.1371/journal.pgph.0004220 (PMC12005593; doi:10.1371/journal.pgph.0004220)
Supplement: S1 Appendix — (DOCX) [file pgph.0004220.s001.docx]

**S1 Appendix**

# **Form 2A: Pre-Exposure Prophylaxis (PrEP) initiation, adherence and service delivery experiences of young Kenyan women**

**In-Depth Interview Guide**

**Target Group**: Young women accessing services within Pamoja CBO community safe spaces.

**Introduction**

I want to thank you for taking the time to meet with me today. My name is ___________________. I am working with the Pamoja Community-Based Organization on a project aiming to learn about experiences with community safe spaces, especially for health services, including PrEP access, uptake, and adherence among young women. In addition, we aim to understand and assess determinants of success across the safe spaces service delivery platforms.

Everything you share during this interview will be kept confidential and will not be shared with your partner, family, or community members afterward. The information that you provide will be used to inform efforts to strengthen and improve health services in Kenya. Remember, you don’t have to talk about anything you don’t want to, and you may end the interview at any time. This interview will not take too much time (around 1.5 hours). If you have questions you want to ask on other topics, I can assist you in finding answers after the interview is over.

(Read the informed consent form aloud and give the participant a copy. If s/he agrees to participate, ask her to sign the informed consent form. This guide includes the topics we will cover and questions that may be helpful in facilitating the interview. You do NOT have to ask all the questions or follow the order given in the guide).

1. **Interview topics with relevant probes.**

Now let us talk about Pre-exposure Prophylaxis (PrEP) and the experience at the community safe spaces under the Pamoja DREAMS program.

First, I would like to understand the process.

- 1. Please tell me more about these safe spaces, especially for PrEP. Who is eligible? How is this eligibility assessed?
  2. What steps would a girl or young woman take to enroll in the program and access a safe space?
  3. How well is the system working to link these women to the health center to get other services? What are the facilitators? What are the barriers?

1. What are the challenges that you have experienced with safe spaces and with PrEP access?
   1. Probe-individuals (defaulters, confidentiality, acceptance), health system (access, HCW attitudes), community (stigma, myths)
2. What are some factors that have made the implementation of the PrEP program easier?
   1. Probe: partnerships, community attitude, safe spaces, interest from girls/young women

**Specific interview questions/probes**

1. How do young women feel about contracting HIV in their lifetime?
2. How many of your friends know about PrEP? Do they want to take it?
3. What are the barriers to accessing PrEP?
4. What other methods of HIV prevention do you or others know and use?
5. How many of your friends know about PrEP? Do they want to take it? What are the barriers? How do they feel about PrEP as an option? What other methods of HIV prevention are they using?
6. What structures are there to support PrEP? What kind of support is available? Probe: safe spaces, Health facilities, community, etc.
7. What challenges do you and other young women face in terms of taking at the safe spaces or elsewhere? Probe – Stigma, access.
8. Describe how you take your services, including PrEP medication, in a safe space. Does this process work well for you? What is your perception about using these services in these safe spaces?
9. What are some things you worry about when accessing safe spaces?
10. What are some things that prevent you from accessing safe spaces?
11. How does taking PrEP and other services at the safe space make you feel? Do you feel more in control of your choices, etc?
12. If you could change something about your access point for PrEP and other services, what would it be?
13. Other community social support system
    1. Other than the Ministry of Health, who else is involved in the service delivery in the safe space?
    2. How do the families, partners, and spouses react to service provision in the safe space, especially for PrEP?: Probe disclosure, stigma, rejection e.t.c
    3. What support system is there for AGYW in the safe spaces? How about in the health sector and in the community at large?
    4. What barriers or challenges do you see with safe spaces and access to services, e.g., PrEP? Probe: (attitudes, knowledge, fear), community, health system (attitudes, stockouts)
    5. What is working for you? Are there follow-up and defaulter tracing mechanisms in the safe spaces?
14. Please describe to me what is in the safe spaces package. Who gets what, and at what point?
    1. How do the services under safe spaces reach the beneficiaries?
    2. How is the information updated? Who updates these services? How often are these services updated?

Thank You!
