# Supplementary material for: Overcoming structural violence through community-based safe-spaces: Qualitative insights from young women on oral HIV pre-exposure prophylaxis (PrEP) in Kisumu, Kenya
Source: PLOS Glob Public Health. 2025 Feb 24;5(2):e0004220. doi: 10.1371/journal.pgph.0004220 (PMC12005593; doi:10.1371/journal.pgph.0004220)
Supplement: S2 Appendix — (DOCX) [file pgph.0004220.s002.docx]

S2 Appendix

Raw dataset for demographic characteristics of young women enrolled in the study

| Participant ID | Age | Marital status | Source of income | Education Level | Religion | Number of children |
| --- | --- | --- | --- | --- | --- | --- |
| IDI 01 | 18 | Single | SME | Form 4 | Christian | 0 |
| IDI 02 | 21 | Single | SME | Class 8 | Christian | 0 |
| IDI 03 | 20 | Single | SME | Class 7 | Christian | 0 |
| IDI 04 | 22 | Married | Farming | College | Christian | 2 |
| IDI 05 | 20 | Married | Farming | Class 8 | Christian | 1 |
| IDI 06 | 19 | Single | SME | Form 1 | Christian | 0 |
| IDI 07 | 20 | Single | SME | Form 4 | Muslim | 0 |
| IDI 08 | 23 | Married | Farming | Class 8 | Christian | 2 |
| IDI 09 | 21 | Single | SME | Form 2 | Christian | 0 |
| IDI 10 | 20 | Single | SME | Class 8 | Christian | 0 |
| IDI 11 | 19 | Single | SME | Class 8 | Christian | 0 |
| IDI 12 | 24 | Married | Farming | Form 4 | Christian | 3 |
| IDI 13 | 23 | Married | Farming | Class 8 | Christian | 3 |
| IDI 14 | 20 | Single | SME | Form 4 | Christian | 0 |
| IDI 15 | 22 | Single | SME | Form 4 | Christian | 1 |
| IDI 16 | 19 | Married | SME | Class 7 | Christian | 1 |
| IDI 17 | 20 | Single | SME | Class 8 | Christian | 0 |
| IDI 18 | 19 | Single | SME | Form 2 | Muslim | 0 |
| IDI 19 | 18 | Single | SME | Form 4 | Christian | 0 |
| IDI 20 | 23 | Married | SME | College | Christian | 2 |
| IDI 21 | 21 | Single | SME | Class 8 | Christian | 1 |
| IDI 22 | 24 | Married | Farming | College | Muslim | 2 |
| IDI 23 | 22 | Single | SME | Form 4 | Christian | 1 |
| IDI 24 | 18 | Single | SME | Class 8 | Christian | 0 |
| IDI 25 | 20 | Single | SME | Class 8 | Muslim | 0 |
| IDI 26 | 18 | Single | SME | Class 7 | Christian | 0 |
| IDI 27 | 21 | Single | SME | Class 8 | Christian | 0 |
| IDI 28 | 20 | Single | SME | Form 4 | Muslim | 0 |
| IDI 29 | 19 | Single | SME | Form 4 | Christian | 0 |
| IDI 30 | 18 | Single | SME | Class 8 | Christian | 0 |
| IDI 31 | 21 | Single | SME | Form 2 | Christian | 0 |
| IDI 32 | 22 | Single | SME | Class 8 | Christian | 0 |
| IDI 33 | 23 | Married | Farming | College | Christian | 2 |
| IDI 34 | 19 | Single | SME | Form 4 | Christian | 0 |
| IDI 35 | 18 | Single | SME | Class 8 | Christian | 0 |
| IDI 36 | 20 | Single | SME | Form 4 | Christian | 0 |
