# Supplementary material for: Overcoming structural violence through community-based safe-spaces: Qualitative insights from young women on oral HIV pre-exposure prophylaxis (PrEP) in Kisumu, Kenya
Source: PLOS Glob Public Health. 2025 Feb 24;5(2):e0004220. doi: 10.1371/journal.pgph.0004220 (PMC12005593; doi:10.1371/journal.pgph.0004220)
Supplement: Attachment — Submitted filename: Response to Reviewers comments-16June2024.docx [file pgph.0004220.s004.docx]

PGPH-D-24-00463
Overcoming structural violence through community-based approaches: Insights from community-based HIV prevention programs in Kisumu, Kenya.
PLOS Global Public Health

**Response to the reviewers’ comments**

Dear Editorial Team,

Thank you for taking the time to review our paper. We are grateful for the substantial feedback from the reviewers. We have carefully read through the comments and incorporated the suggested changes. With these substantial revisions, we are confident that our manuscript now meets the PLOS Global Public Health publication criteria. Therefore, we are happy to submit our revised manuscript for consideration.

Below is a point-by-point response to the comments raised by the reviewers.

3. We have noticed that you have uploaded Supporting Information files, but you have not included a list of legends. Please add a full list of legends for your Supporting Information files after the references list.

*All supporting information now has a list of legends.*

4. Data availability.

*We have provided data on the data points for means, medians, and variance measures for median and IQR in the manuscript.*

5. Review Comments to the Author

Reviewer #1: Thank you for the opportunity to review this manuscript. It's a qualitative paper describing client perspectives on a community based safe space HIV prevention programme and effect/ impact on access to PrEP, adherence to PrEP, stigma and task shifting. The problem with the manuscript is that it's not very clear in its objectives and the descriptions of the interviews generalized as opposed to being specific and some conclusions not supported by the evidence presented. I have listed below specific concerns/ issues I had with the manuscript

- Title: the title refers to community HIV prevention programmes when in reality the authors studied one type of community-based programme in one location i.e. Safe spaces programme in Kisimu

*Thank you for your comment. We have revised the title accordingly, and it now speaks to community safe spaces only. The new title now reads ‘Overcoming structural violence through community-based safe spaces for HIV prevention programs: Insights from Kisumu, Kenya.’*

Abstract
The abstract introduction mentions that HIV prevalence in Kenya decreased to 4.9%. Over what time and from what % baseline. The abstract introduction is not speaking to the title of the paper. I was expecting to read about structural violence among AGYW. At a minimum, I expected the abstract introduction to mention structural violence and its relationship to HIV acquisition and lack of access to HIV care and treatment. The abstract methods doesn't mention what community-based HIV prevention programmes were explored and what informed them. Was there a framework used for the exploration? The conclusion is not speaking to the results presented

*Thank you for your attention to the abstract section of the manuscript. We have noted these concerns and revised the abstract to address these changes. First, we have moved away from the general HIV prevalence rate in Kenya to focus more on adolescent girls and young women. We have also introduced the concept of structural violence in the introduction and expanded on safe spaces and community prevention programs, which is the focus of this paper. We also noted the lack of a framework for exploration in the methods section. We have revised this to include the re-aim framework, which we used in the exploration process. In addition, we have revised the discussion section to align with the results section. See the new abstract on pages 1 and 2.*

Introduction
page 8, lines 8- 11: The issue of HIV incidence among AGYW is more nuanced than this. In SSA, incidence in this group has been going down for many years although it remains highest compared to other risk groups. So, I wouldn't say increased, rather high.

*Thank you for your insights. We have revised this section accordingly to emphasize the “high” instead of the “increase. “*

Methods
page 11, line 110- 114: Why only safe spaces when DREAMS was implemented in many other community-led interventions

Thank you. We realize that we did not provide adequate justification to chosing only safe spaces. We have expanded on this and included additional information as below:

*We chose to examine the community safe spaces because of their unique approaches, including the provision of oral PrEP, contraceptives, and other structural interventions such as financial capability and entrepreneurship. In addition, Pamoja CBO was the only community-based organization offering oral PrEP within the safe spaces at the time of the study.*

Page 12, line 117: provide the equivalent amount in US dollars.

*Thank you. We have revised this section. The new statement now reads as follows:*

*“Each participant was reimbursed up to KES 500 (USD 5) for transportation costs.”*

Page 12, line 138- 142: the authors list the objectives of the in-depth interviews as to explore successes , failures and challenges of Pamoja's community-based HIV prevention and community programmes I think this should be specific to their safe spaces programme since you only interviewed young women attending the safe spaces programme.

*Thank you. We agree with this comment and suggestion. We have thus revised the section to incorporate these changes. It now reads:*

*“We conducted in-depth interviews with female (n=20) participants of different ages to explore the effectiveness of community-driven safe spaces in addressing structural violence in the prevention and treatment of HIV in girls and young women.”*

Page 12, line 142: please include the interview guide used during the indepth interviews.

*Thank you. This has been included as part of the supporting documents under appendix A.*

Page 12, line 144: see comment above regarding HIV prevention programmes when you only spoke to people in the safe spaces programme

*Thank you. We have revised this and narrowed the scope to safe spaces.*

Results
Page 13, line 157: Please present the median and IQR for age

*Thank you. We have made the adjustments and presented the median and the IQR for the age. This section now reads as follows:*

*A total of 36 young women (mean age 20) participated in the research study, with an age range of 18 -24.*

Page 13, line 160: also include the proportion of the young women who were HIV positive and on ART.

*Thank you for this comment, and we are sorry that we did not provide enough context for the DREAMS program. We would like to clarify that the DREAM program under Pamoja CBO only targeted HIV-negative adolescent girls and young women with the aim of preventing HIV infection. We have*

Page 13, Line 163: the authors stated that the study revealed difficulties in accessing health services. Was this specific for HIV related services.

*Thank you for your observation. We are sorry we did not communicate or unpack the sentence clearly to encompass other services. However, other services, such as livelihoods, are addressed in part 3.3 under the sub-title—community-led safe spaces as places for navigating barriers to livelihoods and socioeconomic empowerment.*

Page 14, line 192: this sub-title doesn't reflect what is written below. perhaps add something about adherence

*Thank you for the insights. We have added more information about adherence.*

Page 15, line 224: It seems the clients confused ARVs with PrEP

*Thank you. We have clarified this.*

Page 17, line 283: what sort of needs of the young women were met by the mentors

*Thank you for your comments. We have revised this section accordingly and specified the needs more clearly.*

Discussion
Page 21, line 405: I am not convinced about this task shifting story

*We agree that this was not adequately explored. We have revised this section and moved away from task shifting. Instead, we have re-defined our analysis to focus on the role of community resource persons.*

Page 22, line 421: I think just be specific to HIV prevention as was outlined in the introduction. HIV management covers care and treatment as well

*Thank you. We have narrowed our arguments to HIV prevention only.*
