# Supplementary material for: Overcoming structural violence through community-based safe-spaces: Qualitative insights from young women on oral HIV pre-exposure prophylaxis (PrEP) in Kisumu, Kenya
Source: PLOS Glob Public Health. 2025 Feb 24;5(2):e0004220. doi: 10.1371/journal.pgph.0004220 (PMC12005593; doi:10.1371/journal.pgph.0004220)
Supplement: Attachment — Submitted filename: Response Letter to the Reveiwers Comments-17Aug2024 (1).docx [file pgph.0004220.s005.docx]

PGPH-D-24-00463R1
Overcoming structural violence through community-based safe-space for HIV prevention: Insights from Kisumu, Kenya

Dear Dr. Avanti Dey,

Thank you for taking the time to review our manuscript. We appreciate the reviewers' insights and comments. We have carefully reviewed and incorporated the suggested changes in our revised manuscript, which is attached. See below a point-by-point response to the reviewer’s comments. We feel that the quality of the manuscript has greatly improved and will fully meet PLOS Global Public Health’s publication criteria.

We look forward to a favorable response.

Sincerely,

Patrick Mbullo Owuor,

Corresponding Author

**Response to Review Comment**

Reviewer #1: Thank you for the opportunity to review this revised manuscript. The authors did a good job of addressing the comments I had on the manuscript. I, however still have some more comments based on the information provided

Abstract
Introduction: I thought the authors just looked at HIV prevention, specifically a community-led, safe spaces PrEP program. So, I would recommend removing the care and management from the objectives.

*Thank you. We have now removed care and management from the objective and throughout the manuscript*

Methods: I would make the abstract introduction more succinct and add details about how the study was actually conducted to the abstract methods e.g. study location, study population etc.

We appreciate your careful reading. We have improved the methods section to incorporate the suggested changes. It now reads:

*To explore and assess the effectiveness of community safe spaces in overcoming structural barriers to HIV prevention among adolescent girls and young women, we purposively recruited young women (n=36) enrolled in the Pamoja CBO’s 2022-2023 DREAMS program in Kisumu County, Kenya. Data were collected between June and July 2023 using structured, in-depth interviews (n=20) and focused group discussions (n=16). Guided by The Reach, Effectiveness, Adoption, Implementation, and Maintenance (RE-AIM) framework grounded theory, data were analyzed using Atlas.Ti and organized into themes.*

Results: the authors refer to barriers to structural violence in the abstract results section, this doesn't make sense. I thought the idea was to overcome structural violence and not the barriers to structural violence. When you overcome barriers to structural violence you facilitate the structural violence.

*Thank you. We noted this grammatical error, and we have corrected it as appropriate by removing the word “barrier” from the statement.*

Introduction
Line 60- 61: the authors wrote about eroding progress. Maybe use impeding progress or slowing the progress. There is progress still being made

*Thank you; indeed, we have changed this to “slowing progress” to acknowledge that progress is still being made.*

Line 103: the authors wrote about successes not recognizing CBOs; successes themselves don't recognize CBOs but can lead to greater recognition of the CBOs

*In this sentence, we meant the “roles/efforts made by the community organization not recognized” and not the CBOs. We have revised this for clarity.*

*It now reads:*

*We know that community-led HIV prevention interventions are critical to the fight against HIV (11) and that efforts by community-based interventions have significantly contributed to the milestones in HIV prevention (12). However, documentation of successes in HIV prevention has not adequately captured the roles played by community-based organizations (CBOs).*

Line 108: structural barriers to what?

*Thank you. We have revised it to read:*

*Moreover, despite the role played by CBOs in the fight against HIV, there has been minimal focus on evaluating how community-based HIV prevention interventions have aided vulnerable groups such as young women to overcome structural barriers to HIV prevention.*

Line 135- 136: the authors refer to structural barriers that entrench structural violence within HIV management. shouldn't this be HIV prevention rather?

*Thank you. We have revised this accordingly to focus on HIV prevention and not management.*

Line 137- 138: care and management should be replaced by HIV prevention

*Thank you. We have revised this sentence and removed care and treatment.*

Methods
Line 147: which lake?

*We have noted the oversight and added a reference to Lake Victoria as appropriate.*

Line 207: please refer to HIV prevention as opposed to HIV care and treatment

*Thank you. We have revised this sentence and replaced “HIV care and treatment” with “HIV prevention.”*

Results
Line 221: please include the ages included in the IQR i.e. 19- 22 as opposed to 3

*We have presented the interquartile range (IQR), calculated by getting the difference between Q3 (75th percentile) and Q1 (25th percentile). We have included these as suggested.*

Line 231: remove the word also

*Thank you. We have revised this sentence and removed the word “also.”*

Line 275: disclosure on taking PrEP? I didn't know that there was an issue with disclosure when it comes to taking PrEP. Please elaborate

*We acknowledge that this is a surprising finding since PrEP has been considered to have no disclosure issues. However, our intention here was to report the experiences of the study participants. We have revised the opening of the sentence to reflect this.*

Discussion

Line 375: Please remove the phrase community approaches, such as. The authors only looked at one approach

*Thank you. We have revised this to focus on community-safe spaces.*

Line 376: I would remove the phrase barriers to structural violence. See earlier comment

*Thank you. We have revised and rephrased this sentence.*

Line 379: I am not convinced about this disclosure story

*We have revised our phrasing and acknowledged this as a surprising finding in the population.*

Line 433- 446: since the safe space under consideration focused on PrEP, this is not relevant. Line 461- 464: again, we are talking PrEP, so irrelevant.

*As suggested, we agree with the reviewer’s comments and deleted these two paragraphs from the manuscript.*

Conclusion

Line 512-513: structural barriers to PrEP access? Line 514: shouldn't HIV management be HIV prevention

*Thank you. We have revised the conclusion to take care of these suggestions.*
