# Supplementary material for: Overcoming structural violence through community-based safe-spaces: Qualitative insights from young women on oral HIV pre-exposure prophylaxis (PrEP) in Kisumu, Kenya
Source: PLOS Glob Public Health. 2025 Feb 24;5(2):e0004220. doi: 10.1371/journal.pgph.0004220 (PMC12005593; doi:10.1371/journal.pgph.0004220)
Supplement: Attachment — Submitted filename: PGPH-D-24-00463R2 -Response to Reviewers-Nov 2.docx [file pgph.0004220.s006.docx]

PGPH-D-24-00463R2

**Overcoming structural violence through community-based safe- spaces: Qualitative insights from young women on PrEP in Kisumu, Kenya**

Dear Dr. Annesha Sil

Thank you for reviewing our manuscript, which was submitted for consideration to PLOS Global Public Health. We have extensively revised the manuscript based on the reviewers’ comments, which we found very helpful.

Below, we provide a point-by-point response to the reviewers’ comments.

**Reviewer #2**: Thank you for the opportunity to review this paper on overcoming structural violence through Safe Spaces in Kenya. This is an important topic, however I think there are some major flaws with how the study is at least described. For example, while I do believe in the concept of structural violence, right now, it’s serving more as a distraction to your argument and the tightness of your paper because it’s not consistently described or framed that way, and the results also aren’t framed in that way, leading to some conceptual confusion.

*Thank you for the positive comments on our manuscript. We acknowledge the concept of structural violence was loosely described in our previous version. We have now revised and restructured the paper, making structural violence and its framing consistent throughout the manuscript.*

Also, there are many missing details of the methods so that one cannot adequately assess the quality. The research is described a participatory, but nothing in the methods suggests that is true. And contradictory approaches are described to analysis.

*We do appreciate the attention of the reviewers on the methods section. We have revised the manuscript to include a section on our community engagement and provided details on data collection and analysis.*

I also find the results lacking in a structure that carries the reader through in a logical way and the discussion is not always closely aligned with the results. Additional detailed feedback is available below to help the authors think through these issues further.

*Thank you for your insight helpful insights. We have carefully reviewed our results section and provided relevant quotes to support our findings*

**Abstract**
o The intro talks about structural barriers, but not structural violence so it doesn’t seem aligned with the article title. The reader needs to understand this link between structural barriers and then what you refer to as structural violence (also in the results).
o Why is the RE-AIM theory relevant to this inquiry?
o The way the results are framed, I’m not clear that all the themes came from the young women.

*Thank you. We have revised the abstract to address these concerns. We realized the over-emphasis on structural barriers undermined our statement on structural violence. We have aligned the title and the abstract to focus more on structural violence.*

**• Introduction**
o I see that you do explain your use of structural violence in the introduction, but it comes after you first mention structural violence (line 113) and first mention the study aim. The reader needs to understand your use of this terminology before you mention the aim. I would adjust the introduction to do so. I think you can also do it in less than 2 paragraphs and without as much explanation of only Paul Farmer, but rather introduce that concept, where it came from, what it means, and tie to what we know as structural violence in the AGYW HIV healthcare space.

*Thank you! We have noted the concern and revised the manuscript by introducing and unpacking the concept of structural violence in the second paragraph and before the study aims. See page 5.*

**• Methods**
o You’re missing references from your study location section.

*We provided more context to the study location and added references (17-20) to the study location.*

o I don’t really understand your use of the RE-AIM framework in this participatory qualitative work. That framework feels more useful when doing a proper program evaluation. Also, it feels like it takes away from the described value in community-led knowledge. I actually rather wonder if the concept of structural violence is what you should explain as your study conceptual grounding.

*Thank you for this critical observation. We have carefully re-examined our approach to the data, and we found that the RE-AIM framework was inadequate in this context. Therefore, we resorted to using structural violence as the framework for this study.*

o Typo with your sentence on lines 177 and 178 – double use of ‘program’ – “program beneficiaries of this program”

*Thank you. We have taken care of this and deleted the repeated word.*

o Explain why the threshold of 6-months of participation was deemed meaningful.

*Thank you. We have explained the 6-month participation time inclusion criteria. This section now reads as follows.*

*“Participants included women aged 18 to 24 who had been in the program for over six months. This duration was deemed sufficient for the project beneficiaries to receive essential and primary interventions while attending a safe space. These include evidence-based behavioral interventions (social asset-building skills), structural interventions (socioeconomic approaches), and biomedical interventions (PrEP, contraceptives, post-violence care).”*

o In the Safe Spaces description, you might want to mention the training and support a mentor receives to lead these groups.

*Thank you. We have included additional information on the mentors.*

*“A mentor (usually one of their peers from the same geographical area) manages a safe space. Mentors undergo over five weeks of training on social asset building and work with other community resource persons, including healthcare providers. They schedule activities in the safe spaces and ensure service providers are frequently invited to the safe spaces to talk and provide various services, including sexual and reproductive health services.”*

o Qualitative data collection

♣ Who conducted the IDIs? And I’m unclear why the IDI covered ‘group strategies’ as you mention in line 209. The value of an IDI to cover individual experiences.

*Thank you for your thoughtful observations. We agree with these comments and have provided additional information on IDIs in our revised manuscript. The revised text reads.*

*“Interviews were conducted in individual homes by trained qualitative interviews and lasted approximately one hour. Questions during the interview included individual experiences with cultural and community-based practices that young women must overcome barriers to HIV prevention.”(Page 8)*

♣You don’t sufficiently describe why you conducted FGDs, drawing on the value of FGDs, which is to unpack community level norms, attitudes, experiences. And who conducted those? And how did you decide which participants were in the IDIs vs. FGDs?

*We are very grateful for this technical insight and have revised this section to provide additional information. It now reads*.

*To qualitatively understand and describe the community norms, perceptions, and inter-group dynamics in safe spaces, we conducted two focused group discussions (FGDs) with young women (n=16) from two villages. The FGDs were conducted in identified community spaces, which provided privacy and confidentiality to the participants and lasted about one and a half hours. (page 8-9)*

And were the two groups segmented somehow, similar to how you have varying ages for the IDIs?

*Thank you. The participants for FGDs and IDIs were distinct. However, the groups were not segmented in any age band. We have included this lack of segmentation in our limitations. (Page 19)*

♣ Where were the IDIs and FGDs done?

*Thank you. As highlighted in the above responses, we have revised the respective section e data collection techniques and provided information on where data were collected (pages 8-9).*

*Interviews were conducted in individual homes by trained qualitative interviews and lasted approximately one hour.*

*The FGDs were conducted in identified community spaces, which provided privacy and confidentiality to the participants and lasted for about one and a half hours.*

♣ This section doesn’t adequately describe the structured areas of questioning in both data collection formats.

*Thank you. We have provided broader areas of questioning for both IDIs and FGDs as indicated below (Pages 8-9).*

*IDIS - Questions during the interview included individual perceptions and experiences. For example, we asked participants questions like, “How does taking PrEP and other services at the safe space make you feel? What do you worry about when accessing safe spaces, and how would you describe your personal journey here?*

*FGDs -More broadly, participants were asked about various issues, including cultural norms and practices, especially those concerning how girls and young women are perceived and expected to behave. For example, participants were asked, “How do the families, partners, and spouses react to service provision in the safe space, especially for PrEP?” with specific probing on norms, traditions, family practices, and expectations for young women.*

**o Analysis**

♣ How many people and who was involved in analysis? How was ICR done procedurally?

*Thank you for your insightful comments. We have revised this section to include additional information to address these concerns. See below and also Page 9.*

*“In the first round of coding, the first author (PO) read all the transcripts and generated primary codes while assigning the generated codes to specific segments of the transcripts. In the second round, the second and the third authors (SO and WO) repeatedly went through the transcripts, merging and splitting codes where necessary. In the third and final round, all the researchers reviewed all the transcripts for emerging themes and categorized codes based on similarities of concepts and patterns within the data.”*

♣ How could the analysis be guided by both grounded theory the RE-AIM framework, especially as grounded theory means you let theories emerge from the data? And how was the RE-AIM framework applied? If you did you use, what is the risk in doing so (to be included in the discussion)?

*We realized that the RE-AIM framework did not align with the study design. We have thus dropped this framework and focused on the structural violence framework.*

♣ What was done after coding to analyze the data and come to your interpretations?

Thank you. We have revised this section to highlight how themes were generated and interpreted. See below and page 9.

*“All the researchers reviewed all the transcripts for emerging themes and categorized codes based on similarities of concepts and patterns within the data. Finally, analytic themes were identified using mixed techniques, including scrutiny and word-based techniques (22), starting from frequently used words and searching for subtle concepts in the data.”*

o I’m not seeing anything in your methods to justify calling it participatory action research.

*Thank you. We realized that we overlooked important steps while putting together the manuscript. We have included a section describing our community engagement to address this concern. See below and pages 7-8).*

***Community engagement***

*This study was conducted within the Pamoja Community-Based Organization (Pamoja CBO), a grassroots organization in Kisumu County, Kenya, working to empower communities to identify and address the most significant needs affecting their well-being. The organization has well-established community structures, including community advisory groups (CAGs) as links between the organization and the community. Before data collection, our research team met and worked with representatives from the CAGs to introduce the research and identify and seek permission to engage with the community safe spaces and their participants. In addition, the CAGs met regularly, were appraised of the research activities, and were allowed to ask any clarifying questions throughout the research period.*

**• Results**

o Did you conduct a survey with the qual participants to capture these descriptive characteristics? If so, that should be described in your method.

*Thank you. To collect the demographic characteristics, we provided participants with a log to capture biodata. We have described this under data collection techniques (Page 8) as indicated below:*

*“To help contextualize the data, participants were asked to complete a participant log capturing sociodemographic characteristics, including age, education level, marital status, and number of children at the end of each interview.”*

o What’s a sharing session (line 252)? And I’m not sure the quote exemplifies how the program deconstructed cultural beliefs as it doesn’t speak to prior beliefs.

*Thank you, we have re-examined the data for a more appropriate quote (see below) for this finding.*

*When I started coming to the safe space, my parents were not happy. They said girls are not supposed to go to such spaces on their own. Instead, they wanted me to stay home and help with household chores. They were furious when they heard that we were being taught about safe sex, and they threatened to beat me. I am happy I stayed because I have learned so many things that I did not know. I am also able to get so many services here instead of going to the hospital” (Focused group discussion, 05)*

oThe quote around confidence and disclosure is important, but I’m not sure you’re framing in terms of structural barriers (lines 280-289).Lines 291-301, while important, don’t demonstrate how Safe Spaces overcame stigma. This is more setting the stage for stigma experiences. You could start your results with described structural barriers, including stigma, and then have sections on the role of Safe Spaces in overcoming them. I do feel there needs to be more an understanding of their perceived structural barriers first.o Line 335 – I would remove the HIV positive part of the quote as it doesn’t lend itself to understanding the rest of the quote. o Similar comment as above that lines 342-351 is more about the structural barriers and not about the role of Safe Spaces in navigating them.

*Thank you for your thorough review. Based on these comments, we re-examined the data, provided a more nuanced analysis, and dropped redundant lines. Our findings (pages 10-15) are adequately backed by relevant quotes.*

o Overall, I feel the results could be restructured to reflect first the barriers and then how Safe Spaces were perceived as addressing them.

*Thank you. We have reorganized the entire results section accordingly to incorporate this important observation. Our results section now highlights barriers and explains how safe spaces address them.*

**Discussion**
o The first paragraph of the discussion should summarize the key findings, not just the study approach. Combine the first and second paragraphs.

*Thank you. We have reorganized our results section to incorporate these suggestions. Our first paragraph under discussion (Page 15) summarizes the key findings of the study.*

o I didn’t see in the results anything to support the literacy issue or clearly the task shifting. I also am not sure I saw the women talk about their improved health and well-being or improved relationships with service providers.

*Thank you. We realized we did not provide adequate information on the literacy issue findings. We have now done this by re-examining the data and providing a new explanation in the results section (see section 3.2 of the manuscript). We also cut down our discussion (see page 17) of this concept into a single paragraph.*

*We have also provided new data and additional information on improved provider relationships.*

*“My experience with the clinic has always not been good. I went there when I suspected I had contracted an STI from my abusive partner. Instead, the male clinician I got wanted to take advantage of me and started talking about how he could provide me with better sex and love. Safe space has been different; the health providers who visit our safe space are so friendly; they even visit us at home. They have helped me and other girls a lot.” (In-depth interview,16)*

*However, we have realized we did not provide sufficient evidence on task shifting and therefore opted to omit it in this analysis.*

o I don’t think the health literacy finding as presented in the results is strong enough to warrant two paragraphs about it in the discussion.

*Thank you, we have provided additional results (see section 3.2) with more information while cutting on the length of our discussion on literacy.*

o Overall, I wouldn’t put sub-headings in the discussion and would try to tighten it. Also you should weave reference to your findings into your review and comment of other topics and suggestions. For example with the social support, call attention to what was seen in the data, and then use other literature to describe its importance and future recommendations. You do this a little in the economic empowerment, but your last sentence there is a little weak as I’m not sure what it’s suggesting or recommending as a learning drawing from your work.

*Thank you for these insightful observations. We have addressed this by incorporating the suggestion throughout the discussion section.*

o Line 495, you say you were focused on barriers to PrEP access, which wasn’t the framing earlier.

*Thank you. We have re-aligned this to redirect our focus to structural violence.*
