# Supplementary material for: Overcoming structural violence through community-based safe-spaces: Qualitative insights from young women on oral HIV pre-exposure prophylaxis (PrEP) in Kisumu, Kenya
Source: PLOS Glob Public Health. 2025 Feb 24;5(2):e0004220. doi: 10.1371/journal.pgph.0004220 (PMC12005593; doi:10.1371/journal.pgph.0004220)
Supplement: Attachment — Submitted filename: Response to Reviewers-Nov.25.2024.docx [file pgph.0004220.s007.docx]

Date: November 25, 2024

PGPH-D-24-00463R3
Overcoming structural violence through community-based safe- spaces: Qualitative insights from young women on PrEP in Kisumu, Kenya.
PLOS Global Public Health

Dear

Nancy Angeline Gnanaselvam

Thank you for your feedback and for the opportunity to revise and re-submit our manuscript to PLOS Global Public Health.

We have carefully reviewed the reviewers' comments and revised our manuscript based on their feedback, which we found to be very thorough and relevant. With these revisions, we feel confident that our manuscript will meet PLOS Global Public Health’s publication criteria.

Below, we provide a point-by-point response addressing the points raised by the reviewers.

Sincerely,

Patrick Mbullo Owuor

Corresponding Author

Responses to Reviewers’ Questions

Reviewer #2: Thank you for the opportunity to re-review this paper on a qualitative evaluation of safe spaces and its role in overcoming structural violence among AGYW.

The paper is much improved; however I still had some concerns about the description of the methods as participatory research and also with the strength of the results in how they are presented. My comments are as follows:

**Methods**
o I wouldn’t classify having a CAB as participatory research – it’s community engaged, but not participatory unless they are setting the research agenda, informing the methods, involved in data collection and analysis.

*Response: We appreciate your guidance. We recognize that while our methods were community-oriented, they fell short of fully supporting community-based participatory research techniques. Consequently, we have made revisions to this section. Most notably, we have removed the portion about community-based research.*

**Results**

For the first two themes in results, the presentation of a single quote with one paragraph of text doesn’t lend me to have a sense that these were dominant themes. They feel inadequately supported. Can you give a better sense of their prevalence, so to speak, and some more richness to their presentation? Some of the richness and nuance actually comes through in the section on peer-to-peer education so perhaps you can reorganize. You could describe them as intersecting themes so not try to divide them into three sections and then give description and examples of how they intersect.

*Response: Thank you for your input. We have revised and restructured the results section to incorporate your suggestion. To enhance clarity, we have emphasized the analytic themes and illustrated the connections between these themes and the structural forces that can sustain violence. Please refer to table 2 below:*

**Table 2: Analytic themes on structural barriers to PrEP uptake, related codes, and manifestation of structural violence**

| **Analytic theme** | **Primary code** | **Manifestation of structural violence** |
| --- | --- | --- |
| Institutional barriers | Access gap | Distance, time and privacy |
|  | Provider attitude | Relationship, trust and support |
| Sociocultural barriers | Knowledge gap | Lack of awareness, and poor basic HIV knowledge |
|  | Stigma and disclosure | Worry and fear of being labelled |
|  | Cultural beliefs | Retrogressing gender roles and lack of decision-making powers |
| Economic barriers | Inadequate income | Lack of start-up capital |
|  | Financial literacy | Poor financial skills business planning |
|  | Financial dependency | Male sexual partners |

♣ You might move the provider attitude to a section on healthcare access barriers that are overcome as its related. It could start with a framing of the barriers to healthcare access from a geographic and provider perspective. The provider attitude section currently also feels underdeveloped to stand as a strong theme.

*Thank you. We have revised this section and incorporated these important suggestions by reordering the results section thematically. (See pages 11-12).*

o For the livelihoods theme, the idea of transactional sex comes out of nowhere as it is not mentioned in the introduction and there is nothing beforehand from the qual about it being an issue or connected to structural violence. This could be resolved a bit by framing the start of this paragraph differently and raising transactional sex as a theme among the girls, which was described as being alleviated.

*Thank you for your insights. We have revised this section by first contextualizing transactional sex and then reframing it. (see pages16-17)*

o Generally I think the results feel underdeveloped in a number of areas and may also be strengthened by some re-organization. Is there a way the concept of structural violence could help with that? Does it offer different categories that these could then be ordered in, for example?

*Thank you. We have reframed the results section by illustrating the analytic themes and then laying out several categories (see pages 11-17).*

**Discussion**
o For the stigma paragraph, you may want to review and cite growing literature on PrEP stigma among AGYW. It’s not new that PrEP is associated with ARVs and HIV stigma. See for example:

♣ Hartmann, M., Nyblade, L., Otticha, S., Marton, T., Agot, K., & Roberts, S. T. (2024). The development of a conceptual framework on PrEP stigma among adolescent girls and young women in sub‐Saharan Africa. Journal of the International AIDS Society, 27(2), e26213.

*Thank you for pointing us to this important resource, which we have found very relevant to our work. We have incorporated these new insights into our paper.*
