# Supplementary material for: Overcoming structural violence through community-based safe-spaces: Qualitative insights from young women on oral HIV pre-exposure prophylaxis (PrEP) in Kisumu, Kenya
Source: PLOS Glob Public Health. 2025 Feb 24;5(2):e0004220. doi: 10.1371/journal.pgph.0004220 (PMC12005593; doi:10.1371/journal.pgph.0004220)
Supplement: Attachment — Submitted filename: PGPH-D-24-00463R2 - Response to Reviewers-Dec 19.docx [file pgph.0004220.s008.docx]

**PGPH-D-24-00463R2**

**Overcoming structural violence through community-based safe- spaces: Qualitative insights from young women on PrEP in Kisumu, Kenya**

Dear Nancy Angeline Gnanaselvam

Thank you for reviewing our manuscript, which was submitted for consideration to PLOS Global Public Health. We have extensively revised the manuscript based on the reviewers’ comments, which we found very helpful.

Below, we provide a point-by-point response to the editors’ comments.

     1. A conceptual framework diagram is required to be presented in the article to establish the relationship between the themes and variables

Thank you for this suggestion. We felt a conceptual framework was necessary to help readers understand the complex interplay between the themes and variables of interest. We have illustrated this using the framework shown in Figure 1 on page 10 of the manuscript. Please see below:

Figure 1: A conceptual framework illustrating the role of community safe spaces in overcoming structural violence

**Community Engagement in Safe Spaces**

**Aspects of Violence Addressed**

**Institutional barriers**

-Distance to facility

- Time to facility

- Provider attitude

**Economic barriers**

- Inadequate income

- Financial literacy

- Financial dependency

**IMPROVED**

**WELLBEING**

**Sociocultural barriers**

- Knowledge gaps

- Stigma – HIV & PrEP use

- Cultural practices

**Community safe spaces**

- Biomedical/healthcare services

- Social & cultural connections

- Life skills enhancement

- Economic empowerment

- Awareness & health promotion

**Structural Violence Framework**

**Power & Control**

Economnic violence

sociocultural violence

Violence in healthcare access

Physical violence

Emotional & psychological violence
